# Association between serum calcium concentrations and neuropsychological symptoms of migraine patients in early pregnancy

**DOI:** 10.3389/fneur.2026.1807822

**Published:** 2026-08-06

**Authors:** Huanhuan Gu, Yuanyuan Li, Luyao Zhou, Xiaoying Bi, Jiasi Li

**Affiliations:** 1 Department of Neurology, Changhai Hospital, The First Affiliated Hospital of Naval Medical University, Shanghai, China; 2 Department of Neurology, Changzheng Hospital, The Second Affiliated Hospital of Naval Medical University, Shanghai, China; 3 Department of Neurology, XinHua Hospital, Shanghai Jiao Tong University School of Medicine, Shanghai, China; 4 Department of Neurology, Putuo District People’s Hospital, Shanghai, China; 5 Department of Neurology, Shanghai Mental Health Center, Shanghai Jiao Tong University School of Medicine, Shanghai, China

**Keywords:** early pregnancy, migraine, neuropsychological symptoms, serum calcium, sleep disturbance

## Abstract

**Background:**

Both migraine patients and women in early pregnancy are prone to neuropsychological problems. However, the precise impact of migraine on maternal mental health during early pregnancy is insufficiently comprehended. Calcium is essential for emotional regulation and mental health, with pregnant women being particularly vulnerable to calcium deficiency. It is still uncertain whether serum calcium levels during early pregnancy are associated with the onset or intensity of migraine and neuropsychological symptoms.

**Methods:**

A cross sectional study was used to analyze the differences of neuropsychological symptoms and the serum concentrations of calcium ions, parathyroid hormone, and 25-hydroxyvitamin D in early pregnancy among pregnant women with migraine compared to those without migraine. Furthermore, correlation analyses were performed to evaluate the relationships between the serum concentrations of calcium ions, parathyroid hormone, 25-hydroxyvitamin D and neuropsychological traits within the migraine cohort.

**Results:**

Pregnant women with migraine in early pregnancy exhibited significantly higher somatization symptoms as measured by the Patient Health Questionnaire-15 (PHQ-15). Migraine occurrence was associated with exacerbated emotional disturbances, particularly anxiety and depression, and poorer sleep quality. Furthermore, these women had lower serum calcium levels. Lower calcium levels were associated with greater severity of pain, fatigue, and sleep disturbance items on the PHQ-15. Serum calcium concentrations were negatively correlated with total scores on the Hamilton Depression Rating Scale (HAMD) and the Pittsburgh Sleep Quality Index (PSQI), with particularly strong associations observed for the PSQI subdomains of subjective sleep quality, sleep efficiency, and daytime dysfunction.

**Conclusion:**

Women with migraine in early pregnancy frequently face additional neuropsychological difficulties. A negative correlation was identified between serum calcium levels and diverse neuropsychological symptoms. These findings are correlational and require confirmation in prospective or interventional studies.

## Introduction

Migraine is a highly common primary headache disorder in clinical neurology and is acknowledged as the second foremost cause of neurological disability globally (1). Individuals with migraine often suffer from comorbid conditions, including anxiety, depression, and sleep disturbances, which can significantly impair quality of life (2). The early stages of pregnancy constitute a susceptible phase wherein psychological symptoms—particularly depression, anxiety, and sleep disturbances—are prone to manifest, reoccur, or escalate. Mental health issues compromise the physical well-being of pregnant women and may adversely affect fetal development (3, 4).

Numerous studies have suggested a correlation between migraine and anxiety or depression during the perinatal period. Women experiencing migraine may demonstrate increased emotional sensitivity to adverse stimuli and are more inclined to utilize pessimistic coping strategies (5, 6). However, the precise effect of migraine on maternal mental health in early pregnancy is inadequately comprehended.

Calcium is an essential nutrient integral to various physiological processes, including neurotransmission, cellular signaling, and hormonal regulation, all of which contribute to mental health. Hypocalcemia is linked to a range of neuropsychological symptoms, such as pain, depression, anxiety, sleep disturbances, impaired attention, memory deficits, and fatigue (7). Moreover, several studies have indicated a possible association between reduced serum calcium levels and the etiology of migraine (8).

Pregnant women demonstrate distinct changes in calcium metabolism. To satisfy the physiological requirements of both the mother and the developing fetus, maternal bone resorption escalates, making pregnant women more vulnerable to hypocalcemia. As pregnancy progresses, sufficient calcium consumption becomes increasingly vital for maternal health and fetal development (9). Calcium supplementation for pregnant women is typically commenced after the 14th week of gestation in clinical practice. Migraine symptoms frequently ameliorate after the first trimester. Nonetheless, it is still uncertain if serum calcium levels during early pregnancy are associated with the onset or intensity of migraine and their associated psychological symptoms.

This study seeks to examine the potential correlation between serum calcium levels and psychological symptoms in pregnant women experiencing migraine during early pregnancy. We specifically compared serum calcium concentrations in pregnant women with and without migraine and investigated the correlations between calcium levels and the severity of neuropsychological symptoms within the migraine cohort. The results of this study may improve the early assessment and intervention of neuropsychological symptoms in pregnant women with migraine, thereby facilitating better pregnancy outcomes, minimizing postpartum complications, and enhancing quality of life during the perinatal period.

## Subjects and methods

### Study subjects

This study recruited women in early pregnancy who visited the obstetrics outpatient clinic at our hospital from March 2021 to October 2022. Participants were classified into two groups according to the presence or absence of migraine: the migraine group (women in early pregnancy with migraine) and the control group (age matched women in early pregnancy without migraine). The clinical protocol of the study was approved by the Ethics Committee of the First Affiliated Hospital of Naval Medical University (CHEC2023-092). All participants provided informed consent before enrollment.

Inclusion criteria were as follows:(1) age between 20 and 42 years;(2) within the first trimester of pregnancy (<14 weeks); (3) diagnosis of migraine either prior to pregnancy or newly developed during pregnancy, according to the diagnostic criteria of the International Classification of Headache Disorders, 3rd edition. Women without any history of migraine, either before or during pregnancy, were included in the control group.

Exclusion criteria included: (1) conditions that influence serum calcium levels, including people on medications which may affect calcium metabolism, with a history of primary hyperparathyroidism or pheochromocytoma; (2) presence of serious underlying diseases, including hepatic or renal dysfunction, malignancy, or autoimmune disorders; (3) diagnosis of secondary headache disorders, such as cerebral venous sinus thrombosis.

### Clinical data collection

Demographic and baseline clinical data were obtained from all participants, encompassing age, height, weight, physical activity, duration of sun exposure, and medical history. The questionnaires were conducted according to previous reports (10–13). The intensity of migraine was evaluated using the Visual Analogue Scale (VAS), with scores spanning from 0 to 10. The assessment of migraine-related disability was conducted using the Migraine Disability Assessment Questionnaire (MIDAS) and the Headache Impact Test-6 (HIT-6). Somatic symptoms were evaluated using the Patient Health Questionnaire-15 (PHQ-15), which identifies somatization and classifies severity levels. Anxiety and depression levels were assessed using the Hamilton Anxiety Scale (HAMA) and the Hamilton Depression Scale (HAMD), respectively. The Pittsburgh Sleep Quality Index (PSQI) was used to evaluate sleep quality. Serum concentrations of calcium ions (Ca^2+^), parathyroid hormone (PTH), and 25-hydroxyvitamin D [25(OH)D] were quantified utilizing enzyme-linked immunosorbent assay methodologies.

### Data analysis

Statistical analyses were performed utilizing SPSS version 26.0. Continuous variables exhibiting a normal distribution were presented as mean ± standard deviation, whereas those with a non-normal distribution were expressed as median with interquartile range. Comparisons among groups for continuous variables were conducted using t-test or rank sum test. Covariates in the multivariable linear regression analyses were selected based on clinical plausibility and previously reported associations with both migraine and neuropsychological outcomes. The following variables were considered as potential confounders: gestational age, body mass index (BMI), and serum PTH and 25(OH)D levels. These covariates were entered into the regression models simultaneously. Due to the cross-sectional design of this study, variables such as dietary calcium intake, medication use, and previous psychiatric history were not systematically collected; their potential influence on the observed associations is acknowledged as a limitation (see Discussion). Multivariate linear regression analysis was utilized to assess the relationships between serum Ca^2+^ levels, PTH, and 25(OH)D and neuropsychological symptoms, including anxiety, depression, and sleep disturbances, in pregnant women with migraine. All regression estimates are reported with 95% confidence intervals (CIs) to allow assessment of the precision of the results. A *p*-value of less than 0.05 was deemed statistically significant.

## Results

### Clinical characteristics between groups

The analysis was conducted in a total number of 161 participants (Table 1). The migraine cohort consisted of 57 pregnant women aged 21 to 41 years, with a mean age of 29 ± 4.50 years and a mean gestational age of 11.21 ± 2.12 weeks. The control group comprised 104 pregnant women aged 20 to 42 years, with a mean age of 29 ± 3.50 years and a mean gestational age of 11.65 ± 2.84 weeks. No statistically significant differences were observed between the two groups regarding age, gestational age, body mass index (BMI), years of education, duration of sunlight exposure, or physical activity duration.

**Table 1:** Comparison of general clinical data between two groups.

Variables	Control group (*n* = 104)	Migraine group (*n* = 57)	*F*-value	*p*-value
Age (years)	29 ± 3.50	29 ± 4.50	2.562	0.519
Gestational age (weeks)	11.65 ± 2.84	11.21 ± 2.12	0.316	0.337
BMI (kg/m2)	22.54 ± 3.22	21.77 ± 2.84	1.278	0.237
Years of education (years)	14.00 ± 2.90	14.00 ± 3.40	2.305	0.569
Daily sunlight exposure (minutes/day)	52.05 ± 6.05	47.40 ± 5.02	2.679	0.606
Daily physical activity duration (minutes/day)	94.30 ± 14.73	131.42 ± 22.36	0.004	0.158

### Neuropsychological symptoms between groups

As shown in Table 2, participants in the migraine group exhibited markedly elevated total scores on the Patient Health Questionnaire-15 (PHQ-15) somatization scale compared with the control group. Notably, significantly higher scores were observed in the overall somatization score as well as in the subdomains of pain, pseudo-neurological symptoms, and gastrointestinal distress, whereas no significant differences were found in the cardiovascular, respiratory, sexual, or fatigue subdomains.

Regarding emotional status, the migraine group had significantly higher anxiety and depression scores than the control group, as reflected by increased scores on the Hamilton Anxiety Rating Scale (HAMA) and the Hamilton Depression Rating Scale (HAMD).

**Table 2:** Comparison of neuropsychological symptoms between two groups.

Item	Control group (*n* = 104)	Migraine group (*n* = 57)	*F/Z*-value	*P*-value
PHQ-15				
Total score	5.91 ± 0.42	8.60 ± 0.62	0.057	<0.001
Pain	1 (0, 2)	2 (1.75, 3)	−2.991	0.003
Pseudo-neurological	0.93 ± 0.11	1.43 ± 0.16	0.098	0.008
Cardiovascular	0 (0, 1)	1 (0, 1)	−1.438	0.151
Respiratory	0 (0, 1)	1 (0, 1)	−0.599	0.549
Sexual symptoms	0 (0, 0)	0 (0, 0)	−0.648	0.517
Digestive system	1.40 ± 0.11	2.09 ± 0.15	0.347	0.001
Fatigue	0.74 ± 0.08	0.91 ± 0.09	8.739	0.128
Sleep	0 (0, 1)	0 (0, 1)	−0.870	0.384
HAMA	8.44 ± 0.94	11.97 ± 1.48	0.228	0.039
HAMD	6.52 ± 0.96	9.78 ± 1.02	0.058	0.010
PSQI				
Total	5.10 ± 0.33	6.56 ± 0.51	0.025	0.017
Sleep quality	1 (0, 1)	1 (0, 1)	−1.572	0.116
Sleep latency	1 (0, 1)	1 (0, 2)	−2.568	0.010
Sleep duration	0 (0, 1)	0 (0, 0.5)	−0.964	0.335
Sleep efficiency	0 (0, 0)	0 (0, 0)	−1.000	0.317
Sleep disturbance	1.04 ± 0.05	1.44 ± 0.10	22.079	0.001
Hypnotic drugs	1(0,0)	0(0,0)	−1.394	0.163
Daytime dysfunction	1(0,0)	1.78 ± 0.14	0.565	0.292

In terms of sleep quality, the migraine group showed poorer overall sleep, with a significantly elevated global score on the Pittsburgh Sleep Quality Index (PSQI). Specifically, compared with the control group, the migraine group exhibited significantly higher scores in the sleep latency subcomponent (median [IQR]: 1 [0, 2] vs. 1 [0, 1], *p* = 0.010) and the sleep disturbance subcomponent (mean ± SE: 1.44 ± 0.10 vs. 1.04 ± 0.05, *p* = 0.001). No significant group differences were observed in the other PSQI components, including subjective sleep quality, sleep duration, sleep efficiency, hypnotic medication use, or daytime dysfunction.

### Serum Ca^2+^, PTH, and 25(OH)D levels between groups

As presented in Table 3, serum Ca^2+^ levels were markedly reduced in the migraine group (2.18 ± 0.04 mmoL/L) relative to the control group (2.26 ± 0.01 mmoL/L). Nonetheless, no statistically significant differences were observed between the groups regarding serum concentrations of 25(OH)D or PTH.

**Table 3:** Comparison of serum Ca^2+^, PTH, and 25(OH)D levels between two groups.

Item	Control group (*n* = 104)	Migraine group (*n* = 57)	*F*-value	*P*-value
25(OH)D(nmol/l)	16.07 ± 6.41	16.29 ± 4.93	2.105	0.837
PTH(pg/ml)	25.87 ± 9.55	29.21 ± 16.61	2.697	0.149
Ca^2+^(mmol/l)	2.26 ± 0.01	2.18 ± 0.04	0.057	0.016

### Correlation between serum Ca^2+^ levels and clinical characteristics of migraine in the migraine group

**Figure 1:**
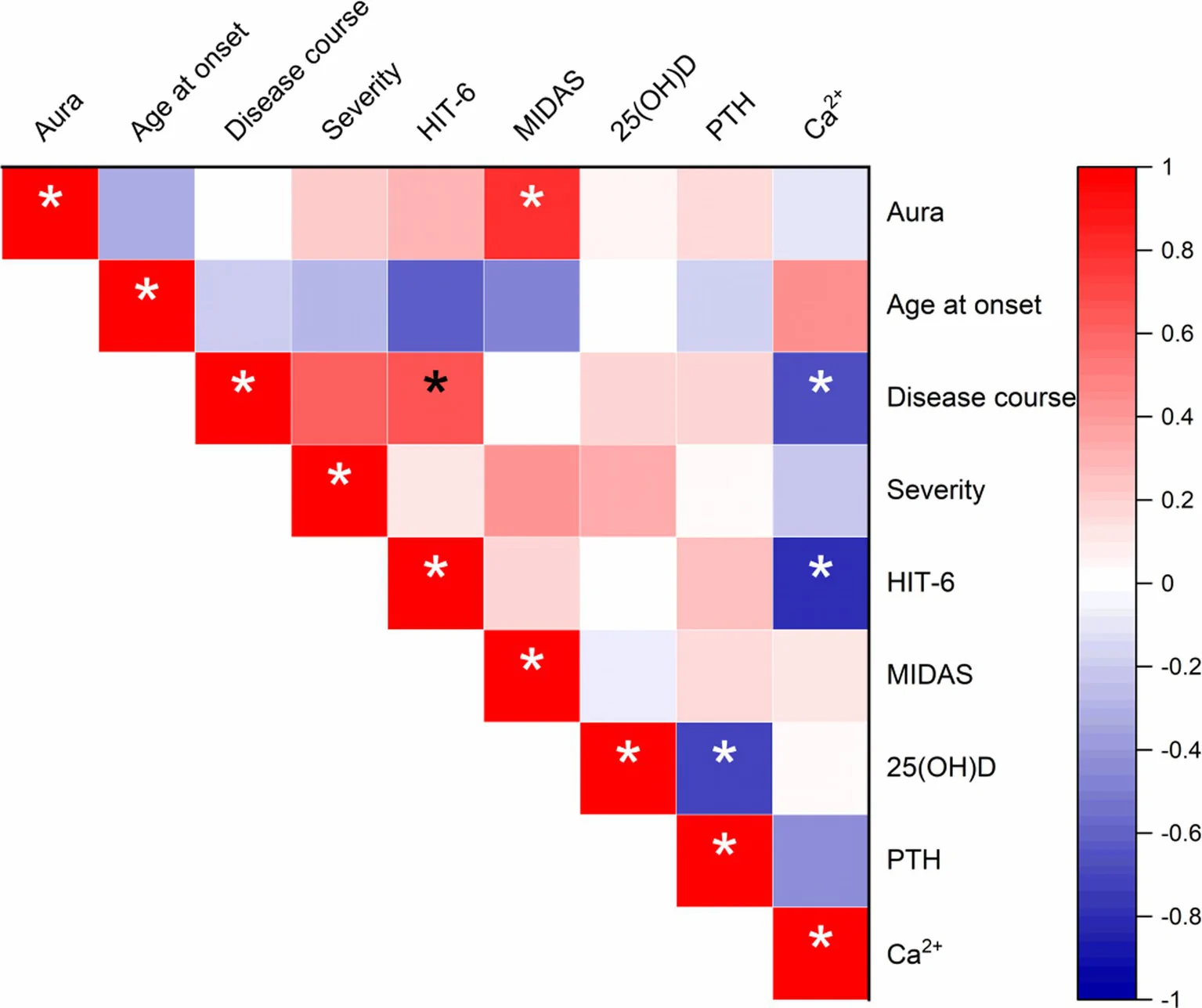
Correlation analysis between serum calcium levels and migraine characteristics in the migraine group.

Figure 1 demonstrated a moderate negative correlation between serum Ca^2+^ levels and the course of migraine (r = −0.526, *p* = 0.038) and HIT-6 (r = −0.621, *p* = 0.043). No significant correlations were found between PTH or 25(OH)D levels and any clinical characteristics of migraine.

### Correlation between serum Ca^2+^ levels and neuropsychological features in the migraine group

As shown in Table 4, a negative association between serum Ca^2+^ levels and multiple neuropsychological indicators in migraine group during early pregnancy was observe. Lower Ca^2+^ levels were associated with more severe symptoms in pain, fatigue, and sleep disturbances as measured by the PHQ-15 somatization scale. Moreover, negative correlations were identified between serum Ca^2+^ concentrations and overall scores on both the HAMD and PSQI, with notably robust associations evident in the PSQI subdomains of subjective sleep quality, sleep efficiency, and daytime dysfunction.

**Table 4:** Correlation analysis between serum Ca^2+^, PTH, 25(OH)D levels, and neuropsychological features in migraine group.

Item	Factors	*β*-value	*Se*	*t*	*P*-value	*95% CI*	*Vif*
PHQ-15							
Total score	25(OH)D	−0.240	0.139	−1.374	0.179	−0.468 to 0.166	1.165
Pain	Ca^2+^	−0.469	0.689	2.218	0.030	−9.829 to 10.946	1.000
Pseudoneurological	25(OH)D	−0.173	0.034	−0.996	0.342	−0.102 to 0.049	0.973
	Ca^2+^	−1.379	2.014	−3.398	0.432	−6.372 to 3.349	1.037
Cardiovascular	Ca^2+^	−0.346	0.003	−2.033	0.053	−1.855 to 4.004	1.251
	PTH	−0.313	0.003	−1.712	0.098	0.003 to 0.044	1.000
Respiratory	PTH	0.380	0.008	2.396	0.077	−0.003 to 0.035	1.045
Sexual symptoms	25(OH)D	−0.134	0.028	0.737	0.468	−0.048 to 0.038	1.118
	Ca^2+^	0.134	0.675	−1.638	0.106	−1.873 to 3.644	1.000
Digestive symptoms	25(OH)D	−0.451	0.029	−2.813	0.008	−0.159 to −0.028	2.834
Fatigue	Ca^2+^	−0.301	1.129	−1.755	0.045	−5.920 to 2.452	2.074
Sleep symptoms	PTH	−0.136	0.009	−2.755	0.456	−0.029 to 0.013	1.097
	Ca^2+^	−0.355	1.445	−1.974	0.048	−5.826 to 0.180	1.097
HAMA	25(OH)D	−0.300	0.306	−1.753	0.090	−1.075 to 0.289	1.069
HAMD	25(OH)D	−0.402	0.211	−2.195	0.079	−0.202 to 0.410	1.177
	Ca^2+^	−0.346	0.193	−0.205	0.046	−64.297 to 23.099	1.247
PSQI							
Total score	PTH	0.029	0.330	0.235	0.815	−0.095 to 0.121	1.028
	Ca^2+^	−0.307	3.088	2.538	0.024	−0.095 to 0.121	1.056
Sleep quality	Ca^2+^	−0.056	0.856	2.036	0.046	−18.900 to 11.961	1.000
Sleep onset latency	PTH	−0.204	0.016	0.354	0.803	−1.761 to 4.446	1.176
	25(OH)D	0.048	0.036	−1.059	0.298	−0.007 to 0.037	1.675
Sleep duration	25(OH)D	0.247	0.034	1.422	0.096	−0.057 to 0.040	1.145
Sleep efficiency	PTH	0.221	0.036	2.017	0.084	−0.023 to 0.080	1.097
	25(OH)D	0.183	0.014	1.429	0.167	−0.029 to 0.018	1.119
	Ca^2+^	−0.224	0.831	1.758	0.048	−3.868 to 2.745	1.077
Sleep disturbance	25(OH)D	−0.243	0.021	−1.397	0.173	−0.076 to 0.021	1.261
Hypnotic drugs	Ca^2+^	0.161	1.070	1.306	0.196	−3.274 to 2.916	1.039
Daytime dysfunction	25(OH)D	−0.366	0.006	2.192	0.036	−0.103 to 0.025	1.287

## Discussion

Of the different forms of acute headache encountered during pregnancy, migraine is the most common. Previous studies suggest that migraine symptoms may ameliorate as pregnancy advances. However, this relief is frequently less noticeable during the first trimester, when migraine attacks may continue or even exacerbate (14–16). During early pregnancy, hormonal variations, challenges in adjusting to physical transformations, and intensified worries regarding maternal and fetal health elevate women’s vulnerability to psychological disturbances, such as anxiety, depression, and sleep disorders (17, 18).

Migraine exhibits a bidirectional relationship with these neuropsychological disturbances, with each condition capable of exacerbating the other. This reciprocal interaction may establish a self-reinforcing cycle that heightens the risk of detrimental maternal and neonatal outcomes (19, 20). Our study indicates, as illustrated in Table 2, that participants in the migraine cohort demonstrated markedly higher total scores on PHQ-15, along with increased subscale scores for pain, pseudo neurological symptoms, and gastrointestinal distress. These individuals exhibited markedly elevated levels of anxiety, depressive symptoms, and sleep disturbances, as indicated by heightened HAMA, HAMD, and PSQI scores, especially in the areas of delayed sleep onset and augmented sleep-related grievances.

The findings indicate that pregnant women suffering from migraine in early pregnancy are more prone to somatization, mood disorders, and diminished sleep quality. Therefore, vigilant neuropsychological surveillance in this demographic is essential. Timely recognition and focused intervention for these symptoms may be crucial in averting the onset or worsening of anxiety and depression, thereby enhancing pregnancy outcomes.

This study confirmed that serum Ca^2+^ levels during early pregnancy are significantly lower in women with migraine compared to those without. Correlation analysis indicated that serum calcium ion levels were negatively correlated with both the course and related disability of migraine. Moreover, inverse correlations were identified between serum Ca^2+^ concentrations and somatization symptoms, including gastrointestinal discomfort, fatigue, and sleep disturbances, as well as with overall scores on the HAMD and the PSQI, encompassing specific subdomains such as subjective sleep quality, sleep efficiency, and daytime dysfunction.

Before discussing the potential implications of these findings, it is important to consider their clinical relevance. The mean difference in PHQ-15 total scores between the migraine and control groups was 2.69 points (8.60 vs. 5.91). A recent systematic review and meta-analysis of 305 studies with 361,243 participants estimated the minimal clinically important difference (MCID) for the PHQ-15 at 3 points (21), suggesting that the observed difference approaches but does not definitively exceed the threshold for clinically meaningful change. For the HAMD, the mean difference between groups was 3.26 points (9.78 vs. 6.52); although this difference was statistically significant, its clinical significance should be interpreted with caution, as formally derived MCID estimates for the HAMD-17 have not been consistently established. Similarly, the PSQI global score difference of 1.46 points (6.56 vs. 5.10) represents a modest but potentially meaningful difference, as scores above 5 are commonly used to define poor sleep quality. These observations suggest that while the associations are statistically robust, their direct translation to clinical practice requires further validation.

Routine prenatal care currently advises calcium supplementation commencing after 14 weeks of gestation to satisfy the nutritional needs of both the mother and fetus (22). However, our findings indicate that women with migraine demonstrate reduced serum calcium levels in early pregnancy compared to those without migraine. The identified negative correlations between serum Ca^2+^ levels and the course and related disability of migraine, and severity of neuropsychological symptoms raise the hypothesis that earlier calcium supplementation might be advisable for this demographic. However, this interpretation must be approached with considerable caution. The present study did not assess dietary calcium intake, calcium deficiency status, supplementation status, or the effects of calcium intervention. Therefore, any recommendations regarding supplementation should be framed as hypotheses for future interventional studies rather than as clinical implications supported by the current data.

The decrease in serum calcium ion concentrations may influence the neuropsychological alterations noted in early pregnancy among women with migraine via several potential mechanisms that have been proposed in the literature: (1) Ca^2+^ are crucial modulators of neuronal excitability. Calcium ions play a key role in stabilizing membrane potential and controlling the excitability of neurons. Calcium deficiency may undermine neuronal membrane stability, reducing the threshold for cortical spreading depression and exacerbating migraine attacks (23). (2) Calcium is essential for sustaining the function of vascular smooth muscle and endothelial cells. A deficiency may lead to cerebral vasospasm or abnormal vasodilation, both of which could precipitate migraine episodes (24, 25). (3) Reduced calcium levels may hinder presynaptic Ca^2+^ influx, thereby obstructing the release of essential neurotransmitters—such as glutamate, serotonin, dopamine, and norepinephrine—associated with the pathophysiology of migraine, anxiety, depression, and sleep disorders (26–30). (4) Calcium deficiency may stimulate glial cells, increase the secretion of pro-inflammatory cytokines, and exacerbate neuroinflammation, thus facilitating central sensitization and intensifying the interplay between pain and emotional dysregulation (31–34).

It is important to emphasize, however, that these mechanistic explanations are derived from previous literature and were not directly examined in the present study. The current findings are correlational and do not establish causation. Moreover, serum calcium is tightly regulated, and peripheral serum concentrations may not directly reflect intracellular calcium signaling or neuronal calcium homeostasis. The relationship between circulating calcium levels and central nervous system calcium dynamics is complex and not fully understood. Discussing this distinction is essential to provide a balanced interpretation and to help readers understand both the strengths and limitations of serum calcium as a biomarker in this context.

Several limitations of this study should be acknowledged. First, the assessment of migraine history was predominantly self-reported, which may introduce recall bias. Second, comprehensive subgroup analyses based on migraine characteristics were not performed. Migraine is a heterogeneous disorder; patients with aura may differ from those without aura in both clinical presentation and underlying pathophysiology. Similarly, chronic migraine differs from episodic migraine in attack frequency and disease burden. Attack frequency, disease duration, and medication use may all influence both neuropsychological symptoms and biochemical markers. These data were not systematically collected in the present study, and this represents an important limitation, as these factors may contribute to variability within the migraine cohort. Third, the current study is a cross-sectional observational study, so the dynamic changes between calcium levels and neuropsychological symptoms remain unclear. The cross-sectional design precludes determination of whether lower calcium contributes to migraine-related symptoms, whether migraine itself influences calcium metabolism, or whether both are influenced by other biological or behavioral factors. Fourth, although we adjusted for several potential confounders, other clinically important variables—including dietary calcium intake, medication use, and previous psychiatric history—were not available. Their potential influence on the observed associations should be considered when interpreting the findings. Fifth, the generalizability of the results is limited by our relatively small sample size. Therefore, larger-scale multicenter studies are required to provide more evidence to determine the ideal timing and dosage of calcium supplementation during early pregnancy.

## Conclusion

In this cross-sectional study, migraine was associated with significant differences in neuropsychological parameters among women in early pregnancy. The most notable manifestation was a heightened occurrence of somatization symptoms, especially pain, pseudoneurological issues, and gastrointestinal complaints. Migraine was associated with higher anxiety and depression scores in early pregnancy, with depressive symptoms exhibiting a more significant escalation. Sleep quality was negatively affected, especially regarding sleep latency and disturbances. Women experiencing migraine in early pregnancy exhibited diminished peripheral serum Ca^2+^ levels, which were inversely correlated with the course and disability of migraine, and the intensity of various neuropsychological symptoms. These associations are correlational and do not imply causation. Prospective longitudinal studies and randomized controlled trials are needed to investigate the dynamic alterations and potential causal effects of calcium levels on neuropsychological symptoms during early pregnancy.

## Data Availability

The raw data supporting the conclusions of this article will be made available by the authors, without undue reservation.
